# Unravelling the mosquito-haemosporidian parasite-bird host network in the southwestern Iberian Peninsula: insights into malaria infections, mosquito community and feeding preferences

**DOI:** 10.1186/s13071-023-05964-1

**Published:** 2023-11-01

**Authors:** Carlos Mora-Rubio, Martina Ferraguti, Sergio Magallanes, Daniel Bravo-Barriga, Irene Hernandez-Caballero, Alfonso Marzal, Florentino de Lope

**Affiliations:** 1 Departamento de Anatomía, Biología Celular y Zoología, Universidad de Extremadura, Facultad de Ciencias, Avenida de Elvas S/N, 06006 Badajoz, Spain; 2 Departamento de Biología de la Conservación y Cambio Global, Estación Biológica de Doñana, EBD-CSIC, Avda. Américo Vespucio 26, 41092 Seville, Spain; 3 Departamento de Sanidad Animal, Parasitología, Universidad de Extremadura, Facultad de Veterinaria, Avda. Universidad S/N, 10003 Cáceres, Spain; 4 Consorcio de Investigación Biomédica en Red de Epidemiología y Salud Pública (CIBERESP), Madrid, Spain; 5 Grupo de Investigaciones en Fauna Silvestre, Universidad Nacional de San Martín, Jr. Maynas 1777, 22021 Tarapoto, Perú

**Keywords:** *Haemoproteus*, Mosquito community composition, Mosquito feeding preference, *Plasmodium*, Vector-borne disease

## Abstract

**Bakground:**

Vector-borne diseases affecting humans, wildlife and livestock have significantly increased their incidence and distribution in the last decades. Because the interaction among vectors-parasite-vertebrate hosts plays a key role driving vector-borne disease transmission, the analyses of the diversity and structure of vector-parasite networks and host-feeding preference may help to assess disease risk. Also, the study of seasonal variations in the structure and composition of vector and parasite communities may elucidate the current patterns of parasite persistence and spread as well as facilitate prediction of how climate variations may impact vector-borne disease transmission. Avian malaria and related haemosporidian parasites constitute an exceptional model to understand the ecology and evolution of vector-borne diseases. However, the characterization of vector-haemosporidian parasite-bird host assemblages is largely unknown in many regions.

**Methods:**

Here, we analyzed 5859 female mosquitoes captured from May to November in five localities from southwestern Spain to explore the composition and seasonal variation of the vector-parasite-vertebrate host network.

**Results:**

We showed a gradual increase in mosquito abundance, peaking in July. A total of 16 different haemosporidian lineages were found infecting 13 mosquito species. Of these assemblages, more than 70% of these vector-parasite associations have not been described in previous studies. Moreover, three *Haemoproteus* lineages were reported for the first time in this study. The prevalence of avian malaria infections in mosquitoes varied significantly across the months, reaching a maximum in November. Mosquito blood-feeding preference was higher for mammals (62.5%), whereas 37.5% of vectors fed on birds, suggesting opportunistic feeding behavior.

**Conclusion:**

These outcomes improve our understanding of disease transmission risk and help tovector control strategies.

**Graphical abstract:**

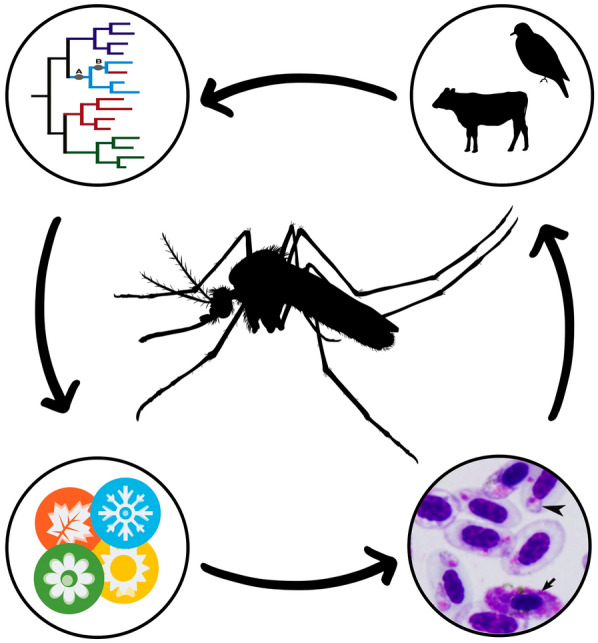

**Supplementary Information:**

The online version contains supplementary material available at 10.1186/s13071-023-05964-1.

## Background

The rise in distribution and incidence of vector-borne diseases in recent decades poses a significant worldwide public health concern with far-reaching economic implications [1, 2]. Mosquitoes (Diptera; Culicidae), with more than 3500 species worldwide, are one of the most important vectors of pathogens, including protozoa (malaria), metazoan (filarial nematodes) and viruses (e.g. West Nile Virus, Dengue) and affect humans, domestic animals and wildlife [3]. The structure and composition of insect vector communities can have profound impacts on pathogen transmission [4]. For example, changes in vector community composition can affect transmission patterns by redistributing disease risk [5, 6]. Therefore, an assessment of the species composition and population dynamics of the local mosquitoes, including mosquito abundance, richness and species diversity, is the crucial step for developing and implementing suitable strategies to control mosquito vector populations that may eventually reduce the spread of deadly mosquito-borne diseases.

The abundance and community composition of mosquitoes are affected by several factors including land use types, landscape transformation and seasonal fluctuations. In this sense, it has been shown that anthropogenic habitat alteration may affect the abundance, diversity and species richness of mosquitoes and also favors the invasion of anthropophilic mosquitoes [7–9]. In addition, changes throughout the year (e.g. seasonality) in vector diversity and abundance are intimately linked to pathogen establishment, persistence, transmission and spread [10]. For example, seasonality would be of importance in determining the phenology of overwintering vector populations and pathogen persistence for one transmission season to the next [11]. Thus, investigation of how vector community structure and composition change over seasons may help to explain current patterns in diseases but also improve our understanding of how climate variations may impact parasite transmission.

Avian malaria and related haemosporidians represent a diverse group of parasites with global distribution that infect birds from many orders [12, 13]. Investigations of bird haemosporidians have historically contributed to important milestones in medical and veterinary parasitology [14]. Nowadays, empirical and experimental studies in avian malaria represent a unique animal model to understand the ecology and evolution of vector-borne diseases [15]. The life cycle of bird haemosporidians requires the involvement of both an insect vector during sexual and sporogonic phases and the blood cells of the avian host for the merogony phase and development of gametocytes [16]. The genus *Plasmodium* encompasses approximately 40 recognized species, while the genus *Haemoproteus* comprises 130 species, and the genus *Leucocytozoon* has 35 recognized species [12]. Each of these genera has its own specific vectors, with only *Plasmodium* parasites being transmitted by mosquitoes [12]. However, an increasing number of studies have reported infections of *Haemoproteus* in these insect vectors, suggesting their potential competence for transmitting this genus of parasites [17–19].

Characterizing the diversity and structure of vector-parasite networks is crucial to understanding their eco-evolutionary dynamics and disease transmission risk. The transmission of malaria parasites by blood-sucking insects to humans and other animals depends on vector-host interactions [20]. These interactions between blood parasites and their vectors are a complex process influenced by genetic and ecological factors, leading to spatial and temporal variation in parasite prevalence across the distribution of the vectors [21–24]. Moreover, these vector-parasite interactions that determine the disease transmission may be highly specific [25]. For example, the impact of parasite identity and mosquito species on the transmission rate and survival costs of avian *Plasmodium* infections in vectors has been examined [26], showing that avian *Plasmodium* transmission differs among different mosquito species and haemosporidian lineages. Also, vector competence can vary among species and populations [27], depending on a pathogen's ability to develop inside the insect and the mosquito's capacity to generate effective immune responses [28]. Comparative studies across different sites and latitudes have shown a great diversity of haemosporidian parasites in birds from southwestern Iberian Peninsula [14, 29]. Nonetheless, more than 70 haemosporidian lineages have been found infecting birds from this region (MalAvi database version 2.5.7, accessed on August 5, 2023). In addition, 36 mosquito species belonging to six genera have been described in southwestern Spain [24, 30]. However, the characterization of mosquito vector-haemosporidian parasite assemblages remains unknown for this region.

Identifying the factors influencing the distribution, diversity and structure of parasite assemblages is crucial to understand host-parasite dynamics and disease transmission risk [31]. Differences in temporal distributions of the parasites in vectors and vertebrate host species can result in the absence of establishment of interactions among them. For example, Inumaru et al. [32] investigated the prevalence of avian malaria and related haemosporidian parasites in both penguins and mosquitoes at an aquarium in northern Japan across multiple years, showing a mismatch in parasite composition between penguins and mosquitoes. Also, Gangoso et al. [33] analyzed the parasite transmission network in an insular system formed by Eleonora's falcon (*Falco eleonorae*) as avian host, louse flies that parasitize the falcons as potential vectors and avian haemosporidians, showing a mismatch between the malaria lineages isolated in adult falcons and those found in louse flies. Thus, analyzing seasonal variation in the prevalence of haemosporidian parasites in the mosquito vectors will help to predict distributional patterns and to reveal vertebrate host-parasite-vector networks. For example, the detection of infected mosquitoes during late autumn and winter would support the possibility of avian malaria parasites overwintering through infected females. Moreover, because the distributions of avian haemosporidian parasites can vary at macro and local scales [34–36], specific data are required for understanding the assembly of vector-host communities in a particular region. For example, Neto et al. [29] assessed the seasonal variation in prevalence of haemosporidian parasites in house sparrows (*Passer domesticus*) sampled across 1 year at four temperate European sites spanning a latitudinal range of 17°C, showing that seasonality in malaria prevalence is site-dependent, being more pronounced in Spain; specifically, sparrows from SW Spain showed a lower probability of malaria infection in the winter months and then increased progressively until reaching a peak in late summer. However, whether these seasonal differences in infection probability of malaria in vertebrate hosts are accompanied by monthly variation in haemosporidian prevalence within mosquito vectors in this region is unclear.

The interaction between vectors and their hosts plays a key role driving vector-borne disease transmission [37]. The transmission network is ultimately influenced by mosquitoe feeding behavior, which regulates contact rates between infected and susceptible vertebrates [38, 39], thus determining insect infection patterns. Host choice and blood-feeding behavior of mosquito vectors are key parameters in malaria epidemiology because they can influence important features determining vectorial potential, such as feeding rates, adult survival, hatching rates and fecundity of the mosquitoes [40, 41], thus affecting the spatial distribution of the disease [42, 43]. Also, the study of host selection behavior by vector organisms is crucial to recognize reservoir hosts for vector-borne zoonotic pathogens [44, 45]. Therefore, the identification of the source of blood meal in mosquitoes is of prime importance for understanding the transmission dynamics of vector-borne diseases and to design improved vector control strategies [46]. Nineteen mosquito species, potential vectors of important pathogens of medical and veterinary relevance, have been recently identified in the studied area [30]. However, little is known about the host choice of these vector species; therefore our knowledge into possible parasite transmission and zoonotic pathogen spill over/spillback is still limited.

Here, we investigated the composition and seasonally variation of the vector-parasite-vertebrate host network to provide insight into the transmission risk of avian malaria. Specifically, we first aimed to explore the seasonal variation in richness, abundance and diversity of mosquito vectors in southwest Iberian Peninsula. Second, we also analyzed the prevalence and genetic diversity of avian malaria lineages in mosquito vectors to reveal vector-parasite associations. Third, we studied the variations across months of avian malaria prevalence in mosquito vectors. Finally, we explored the relationship between mosquito species and common hosts to decipher host choice through the identification of blood meal sources in mosquitoes.

## Methods

### Study area and sample collection

**Fig. 1:**
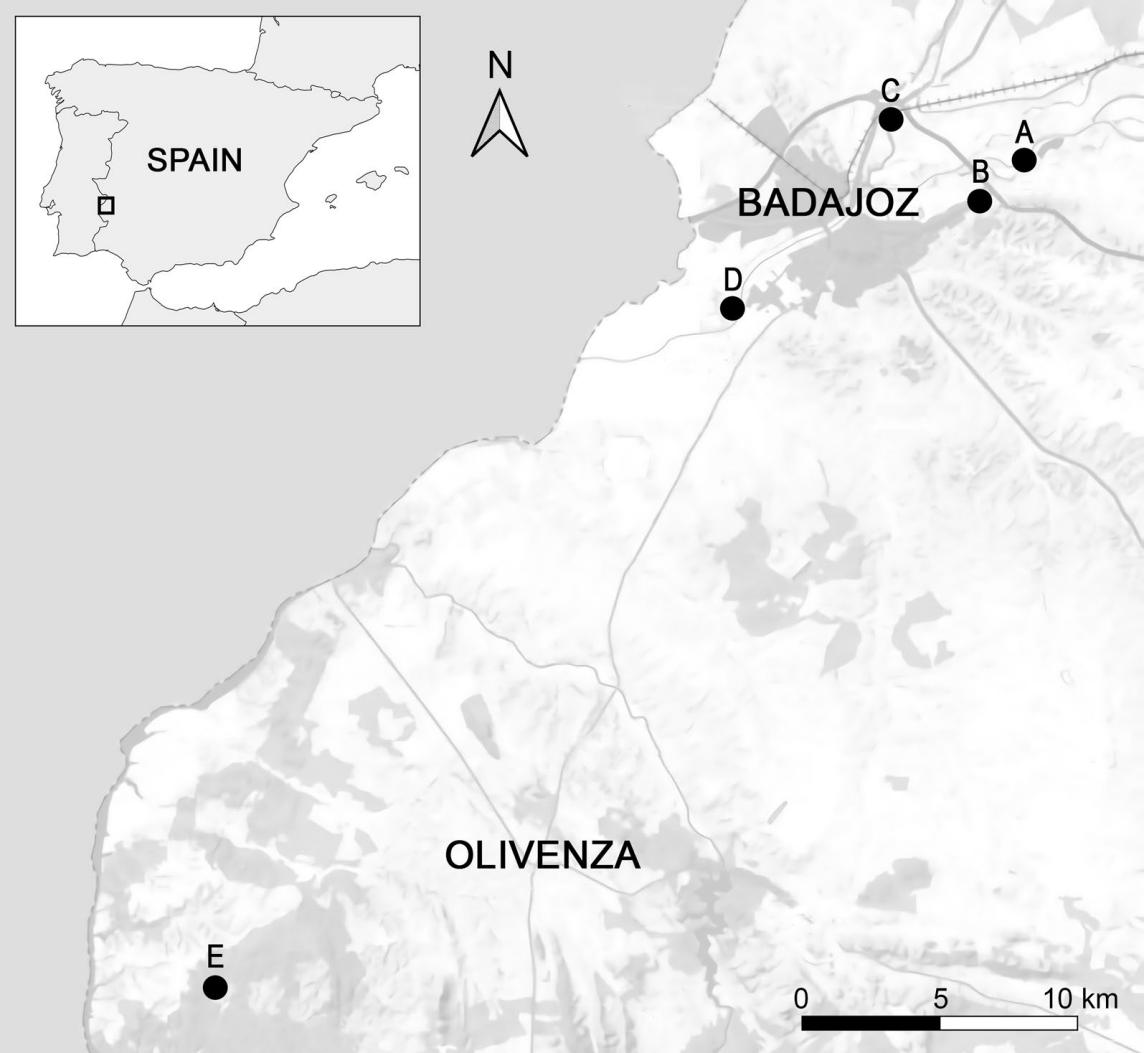
Distribution of the mosquito sampling sites. This map was created using QGIS 3.14.16 (**A**: Sagrajas; **B**: Bótoa; **C**: Gévora; **D**: Azud; **E**: Asesera)

Mosquitoes were captured from May to November 2020 at five sampling sites in the Badajoz and Olivenza municipalities of the Extremadura region (southwestern Spain, Fig. 1). Most of the areas were located close to the border with Portugal and the Guadiana River. Overall, Extremadura has a Mediterranean climate, characterized by a long dry summer season and higher levels of precipitation in winter, according to the Köppen climate classification [47]. Insects were captured using BG-Sentinel and Center for Disease Control (CDC) incandescent light-traps baited with dry ice as source of CO_2_ and gravity traps baited with a hay infusion prepared by incubating 0.5 kg of hay in 114 l tap water for 5 days [48, 49]. Five sampling sessions were conducted at each site, resulting in a total trapping effort of 25 trapping nights. All traps remained active for an average duration of 15 h per capture session, starting between 5:00 and 9:30 p.m., with a frequency of once every 40 days. Insect samples were preserved in dry ice and stored at − 80 °C until identification. Frozen mosquitoes were separated by gender and feeding status over a filter paper on a Petri plate on a chill table. Blood-fed females were identified visually by their dilated red abdomens and stored individually at − 20 °C until subsequent blood meal analysis. Unfed females were grouped entirely in pools (including head, thorax and abdomen) containing from one to 25 mosquitoes according to species, sampling locality and date of collection.

### Morphological and molecular mosquito identification

Morphological identification was performed under a stereomicroscope using appropriate taxonomic keys ([50]; *MosKeyTool* software https://www.medilabsecure.com/moskeytool). The organization and nomenclature of mosquito species were based on two sources: the Systematic Catalog of Culicidae [51], which provided a general framework for the taxonomy of mosquitoes, and Wilkerson et al. [52], which was used specifically for the tribe Aedini. However, due to the difficulty in morphologically identifying *Culex univittatus* and *Cx. perexiguus* [30], some specimens were genetically identified using the primers LCO1490 (5’GGTCAACAAATCATAAAGATATTGG3’) and HCO2198 (5’TAAACTTCAGGGTGACCAAAAAATCA3’) to amplify a ~ 658-bp fragment of the COI gene with a modified PCR thermal cycle [53]. Nucleotide sequences were deposited at DNA Data Bank of Japan (https://www.ddbj.nig.ac.jp/index-e.html) under the accession numbers LC659916 − 8.

Molecular identification of the *Cx. pipiens* complex was carried out by a multiplex PCR assay targeting polymorphisms in the intron-2 of the acetylcholinesterase-2 (Ace-2) gene [54]. Specific primers were used to identify *Culex pipiens* s.s., *Cx. quinquefasciatus*, and *Cx. torrentium*. To differentiate between *molestus* and *pipiens* forms, a PCR amplification of the flanking region of microsatellite CQ11FL was performed [55]. In addition, species identification of four members of the *Anopheles maculipennis* complex (*An. atroparvus*, *An. labranchiae*, *An. maculipennis* s.s. and *An. melanoon*) was performed by a PCR-RFLP assay targeting polymorphisms in the Internal Transcribed Spacer 2 (ITS-2) of the ribosomal DNA [56]. Amplicons from both PCR methods were separated by 2% agarose gel electrophoresis and with a 100-bp DNA ladder as a molecular weight marker (GeneRuler 100 bp DNA Ladder; Thermo Fisher Scientific).

### Molecular detection of haemosporidian infection

DNA samples were extracted from mosquito pools using MAGMAX PATHOGEN RNA/DNA Kit (Applied Biosystems™, reference: 4,462,359). Genomic DNA, diluted to a concentration of 25 ng/μl, was used as a template in a nested polymerase chain reaction (nested-PCR) to determine the presence or absence of haemosporidian infections in the collected vectors, using the protocols described by Hellgren et al. [57]. The amplification was evaluated by running 2.5 µl of final PCR product on a 2% agarose gel. All PCR experiments contained one negative control for every eight samples.

PCR products linked to positive amplifications were purified and sequenced on an ABI 3130 genetic analyzer (provided by the Service of Bioscience Applied Techniques of the University of Extremadura, SAIUEx). The obtained 478-bp sequences of the *cyt-b* were aligned and edited using Geneious software [58]. The final sequences were compared to those in the MalAvi database (version 2.5.7, August 5, 2023, [59]) to identify the parasite lineage. Parasites with sequences differing by one nucleotide substitution were considered to represent evolutionary independent lineages [60, 61]. The nucleotide sequences obtained from new lineages were deposited at DNA Data Bank of Japan (https://www.ddbj.nig. Ac.jp/index-e.html).

### Determination of insect blood meal sources

Blood-fed mosquitoes were screened for the presence of DNA from vertebrate hosts in their blood meal. The abdomen of individual engorged mosquitoes was excised using sterile tweezers, and DNA was extracted using MAGMAX PATHOGEN RNA/DNA KIT (Applied Biosystems™, reference: 4,462,359). The identification of blood meal sources was accomplished using the protocol outlined in Alcaide et al. [62]. This method employed a nested PCR approach using the primary pair of primers BCFW-M13 (5’TGTAAAACGACGGCCAGTHAAYCAYAARGAYATYGG3’) and BCRV1 (5’GCYCANACYATNCCYATRTA3’) and the nested primer pair M13 (5’GTAAAACGACGGCCAGTG3’) and BCRV2 (5’ACYATNCCYATRTANCCRAANGG3’) [62]. Sequences were edited using the Geneious software and identified by comparison with BLAST to assign unknown COI sequences to particular vertebrate species. Host species assignment was considered completed when we found a match ≤ 99% between our sequences and those in GenBank.

### Phylogenetic and statistical analyses

We selected 30 sequences from the 75 positive samples for phylogenetic reconstruction, representing all *Plasmodium* and *Haemoproteus* lineages found. The sequences were aligned using the CLUSTALW algorithm implemented in MEGA11 [63], and a fragment length of 478 pb was chosen for further analyses. Maximum likelihood (ML) optimization criterion was used for phylogenetic reconstruction of *Plasmodium* and *Haemoproteus* lineages assuming the GTR + F + I + G4 model as defined by IQ-TREE [64], considering the Akaike information criterion. The topological support of the branches in the trees was assessed with bootstrap analysis and an approximate likelihood ratio test (aLRT) in Iqtree. In either case, 1000 replicates of the original sequence data were used, and bootstrap or aLRT values ≥ 75% were considered as indicating strong topological support. The obtained trees were visualized using FigTree v1.4.2 (http://tree.bio.ed.ac.uk/software/figtree/).

To estimate the prevalence of blood parasites in mosquitoes, EpiTools software available from AusVet Animal Health Services (https://epitools.ausvet.com.au/) was used, considering differences in pool size and assuming 100% sensitivity and specificity [65]. We estimated whether the prevalence varied over the seasons by using a Pearson’s Chi-squared test followed by post hoc analysis to determine the months when the prevalence of blood parasites was the highest.

Two linear models were used to analyze the mosquito community, incorporating the following continuous dependent variables calculated for each month: (i) total abundance of mosquitoes, assessed as the cumulative count of female mosquitoes belonging to each captured species; (ii) mosquito richness, estimated by the rarefaction index (hereafter referred to as ‘richness’), to account for variations in the number of samples collected across different months [66]; (iii) mosquito diversity, measured by estimating the Shannon index [67]. Estimated marginal means (by *emmeans* function) were conducted to explore variation in mosquito abundance among the different months. All statistical analyses were performed using R software version 4.2.2.

## Results

### Mosquito species composition and phenology

**Fig. 2:**
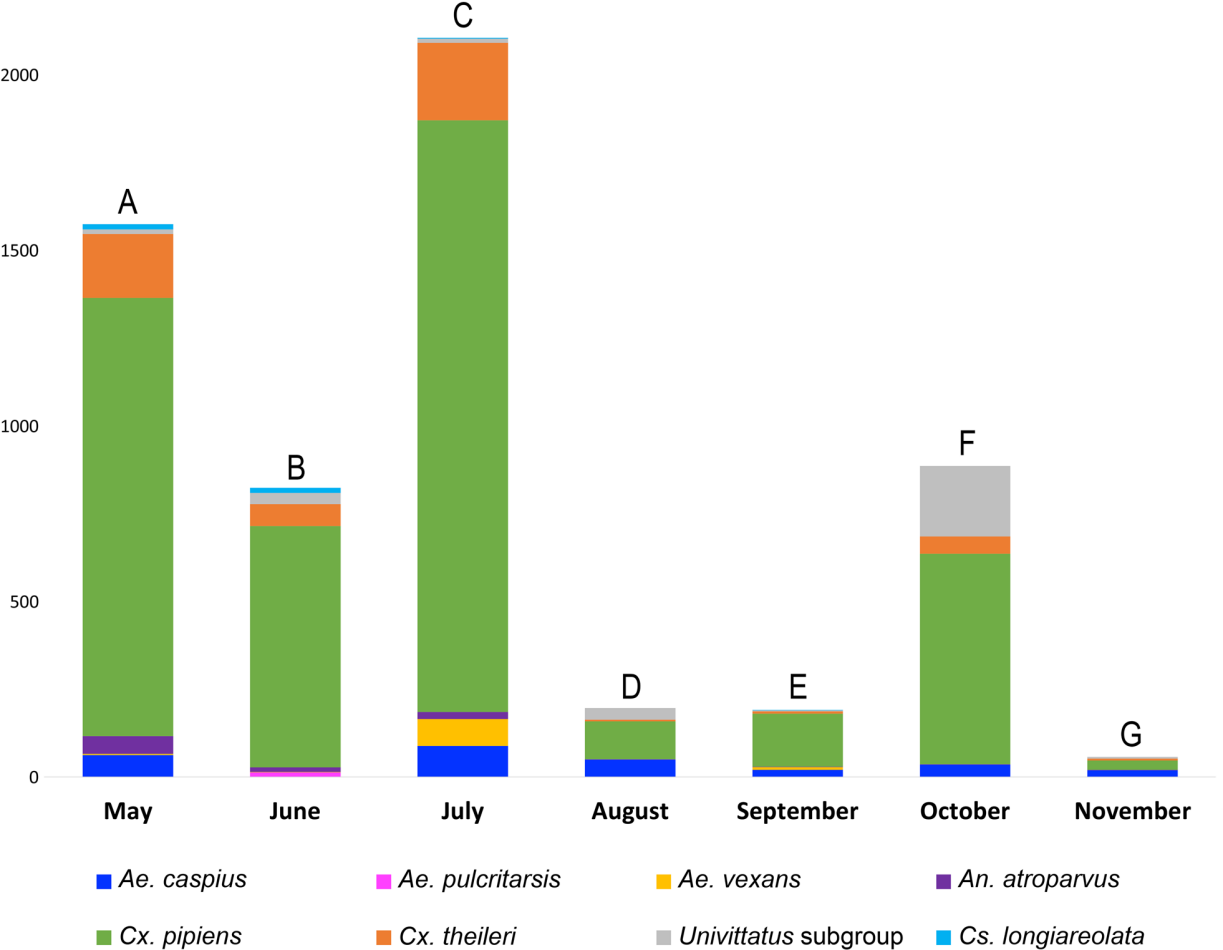
Mean number of female mosquitoes captured per month. Only species with more than 10 individuals are shown. Note that a statistically significant variation in mosquito abundance among months was observed, with the following comparisons showing significant differences: **A** > **E**, **B** > **E**, **C** > **D**, **C** > **E**, **C** > **G** and **E** < **F**

Overall, 5859 female mosquitoes were collected. Thirteen different mosquito species were identified, including *Culex pipiens* (*n* = 4,508), *Cx. theileri* (*n* = 531), *Univittatus* subgroup (consisting of both *Cx. perexiguus* and *Cx. univittatus* found in the studied areas) (*n* = 298), *Aedes caspius* (*n* = 273), *Ae. vexans* (*n* = 90), *Anopheles atroparvus* (*n* = 83), *Culiseta longiareolata* (*n* = 32), *Cs. annulata* (*n* = 14), *Ae. pulcritarsis* (*n* = 13), *Ae. berlandi* (*n* = 10), *Ae. echinus* (*n* = 4) and *Cs. subochrea* (*n* = 3). The average species richness of captured mosquitoes per month was eight species (ranging from five to 10), the average richness of mosquito species was 2.82 (ranging from 2.46 to 3.58), and the average mosquito diversity was 0.929 (ranging from 0.755 to 1.284). No significant differences were found between the richness and diversity values and the trapping months (*P*-values > 0.05). However, mosquito abundance significantly varied with the season, with a gradual increase from May, peaking in July, and then declining in August and September (see Additional file 1: Table S1; Fig. 2). A second peak of abundance was also observed in October.

### Prevalence and genetic diversity of haemosporidian parasites in unfed and blood fed mosquitoes

Overall, 406 pools of unfed mosquitoes were screened for the presence of blood parasites. Of them, 78 pools were positive to at least one parasite genus. In two pools of *Cx. pipiens* we were not able to identify the parasite lineage, while six pools showed evidence of double infections (four pools of *Cx. pipiens*, one of *An. atroparvus* and one of the *Univittatus* subgroup), which made it difficult to identify any particular lineage due to the presence of double peaks in the spectropherograme. In addition, four of 27 blood-fed *Cx. pipiens* showed a positive amplification of malaria parasites.

The highest prevalence for *Plasmodium* was found in *Ae. caspius* and *An. atroparvus*, while *Cs. annulata* and *Cs. longiaerolata* showed the highest prevalence for *Haemoproteus*. Parasite prevalence for each mosquito species is shown in Table 1.

**Table 1:** Prevalence and 95% confidence limits (CL) of avian malaria parasites in mosquitoes, estimated using *EpiTools*

Parasite genus	Mosquito species	No. pools	Range	Positive pools	Prevalence (%)	Lower 95% CL	Upper 95% CL
*Plasmodium*	* Aedes berlandi*	6	1–3	0	0	0	0
	* Ae. caspius*	29	1–25	2	0.74	0.12	2.27
	* Ae. echinus*	1	4	0	0	0	0
	*Ae. pulcritarsis*	2	6–7	0	0	0	0
	*Ae. vexans*	13	1–25	0	0	0	0
	*Anopheles atroparvus*	28	1–21	3	4.53	1.13	11.70
	*Culex pipiens*	250	1–25	36	0.87	0.61	1.18
	*Cx. theileri*	48	1–25	0	0	0	0
	* Univittatus* subgroup	33	1–25	3	1.06	0.26	2.73
	*Culiseta annulata*	8	1–6	0	0	0	0
	*Cs. longiareolata*	14	1–11	0	0	0	0
	*Cs. subrochea*	3	1	0	0	0	0
*Haemoproteus*	*Aedes berlandi*	6	1–3	0	0	0	0
	*Ae. caspius*	29	1–25	2	0.79	0.13	2.41
	*Ae. echinus*	1	4	0	0	0	0
	*Ae. pulcritarsis*	2	6–7	0	0	0	0
	*Ae. vexans*	13	1–25	0	0	0	0
	*Anopheles atroparvus*	28	1–21	3	3.51	0.88	8.85
	*Culex pipiens*	250	1–25	22	0.53	0.34	0.78
	*Cx. theileri*	48	1–25	6	1.21	0.48	2.43
	*Univittatus* subgroup	33	1–25	2	0.69	0.11	2.11
	*Culiseta annulata*	8	1–6	1	7.14	0.42	27.86
	*Cs. longiareolata*	14	1–11	2	6.25	1.07	18.07
	*Cs. subochrea*	3	1	0	0	0	0

The term “Range” denotes the lowest and highest counts of mosquitoes included in the pools for each species

Overall, 16 different haemosporidian lineages were found (nine *Plasmodium* and seven *Haemoproteus)* of which 13 completely matched with previously described lineages, and three *Haemoproteus* lineages were described for the first time (CXPIP34, CXPIP35 and CXPIP36, Table 2). Our findings also revealed 70.3% new vector-parasite associations, with 19 of 27 vector-parasite assemblages described for the first time in this study (Table 2). *Haemoproteus minutus* TURDUS2 and *Plasmodium vaughani* SYAT05 were the most prevalent lineages as they were found in a higher number of vector species. The remaining parasite lineages were isolated in fewer than four pools, and they were only found in one or two vector species (Table 2).

**Table 2:** Avian *Plasmodium* and *Haemoproteus* lineages detected in female blood-fed individuals and unfed mosquito pools. Information on the previous vector species in which lineages were described was extracted from the “Vector data table” in MalAvi database (version 2.5.7, accessed on August 5, 2023)

Parasite genus	Parasite morphospecies	Parasite lineage	Sample size	Locality	GenBank no.	Vector species from our study	Vector species from literature
*Plasmodium*	*Plasmodium* sp.	CXPER01	1	C	JX975222^1^	Culex pipiens^*^	*Culex neavei* *Cx. perexiguus* *Cx. perfidiosus*
	*Plasmodium* sp.	CXPIP23	4	C, E	JX458333^2^	*Culex pipiens*	*Aedes caspius* *Culex pipiens*
	*Plasmodium* sp.	DELURB5	2	A, D	EU154347	Anopheles atroparvus^*^ *Cx. pipiens*	*Culex perexiguus* *Cx. pipiens* *Cx. theileri*
	*Plasmodium* sp.	DONANA02	1	D	JX458327	Culex pipiens^*^	*Culex modestus*
	*P. matutinum*	LINN1	4	B, D, E	DQ847270	*Culex pipiens*	*Aedes caspius* *Culex hortensis* *Cx. modestus* *Cx. perexiguus* *Cx. pipiens* *Cx. restuans*
	*Plasmodium* sp.	PADOM1	1	D	DQ058611	*Culex pipiens*	*Culex pipiens*
	*P. relictum*	SGS1	1	D	AF495571	*Culex pipiens*	*Aedes albopictus* *Culiseta annulata* *Culex modestus* *Culex pipiens* *Culex sasai* *Culex theileri* *Culex perexiguus* *Lutzia vorax*
	*P. vaughani*	SYAT05	21	A-E	DQ847271	Anopheles atroparvus^*^ Aedes caspius^*^ *Culex pipiens* *Univittatus* subgroup	*Aedes albopictus* *Culex modestus* *Culex perexiguus* *Culex pipiens* *Culex restuans* *Culex theileri*
	*Plasmodium* sp.	SYAT24	3	D	AY831749	Culex pipiens^*^	*-*
*Haemoproteus*	*Haemoproteus* sp.	CXPIP34	1	E	LC743560	Culex pipiens^*^	*-*
	*Haemoproteus* sp.	CXPIP35	1	D	LC743559	Culex pipiens^*^	*-*
	*Haemoproteus* sp.	CXPIP36	1	D	LC743558	Culex pipiens^*^	*-*
	*Haemoproteus* sp.	PHSIB1	1	D	AF495565	Culex pipiens^*^	*-*
	*Haemoproteus* sp.	RW4	1	D	KY768830	Culex theileri^*^	*-*
	*H. minutus*	TUPHI1	4	D, E	GU085191	Culex pipiens^*^ *Univittatus* subgroup^*^	*-*
	*H. minutus*	TURDUS2	27	A-E	DQ060772	Anopheles atroparvus^*^ Aedes caspius^*^ Culiseta annulata^*^ Culiseta longiareolata^*^ *Univittatus* subgroup^*^ *Culex pipiens* Culex theileri^*^	*Culex pipiens*

^1^Accession number refers to the lineage synonyms DONANA10 described in MalAvi (version 2.5.7, August 5^th^, 2023)

^2^Accession number refers to the lineage synonyms DONANA09 described in MalAvi (version 2.5.7, August 5^th^, 2023)

Asterisks (*) denote new associations between haemosporidian lineage and vector species described for the first time in this study. Localities: A: Sagrajas; B: Bótoa; C: Gévora; D: Azud; E: Asesera

**Fig. 3:**
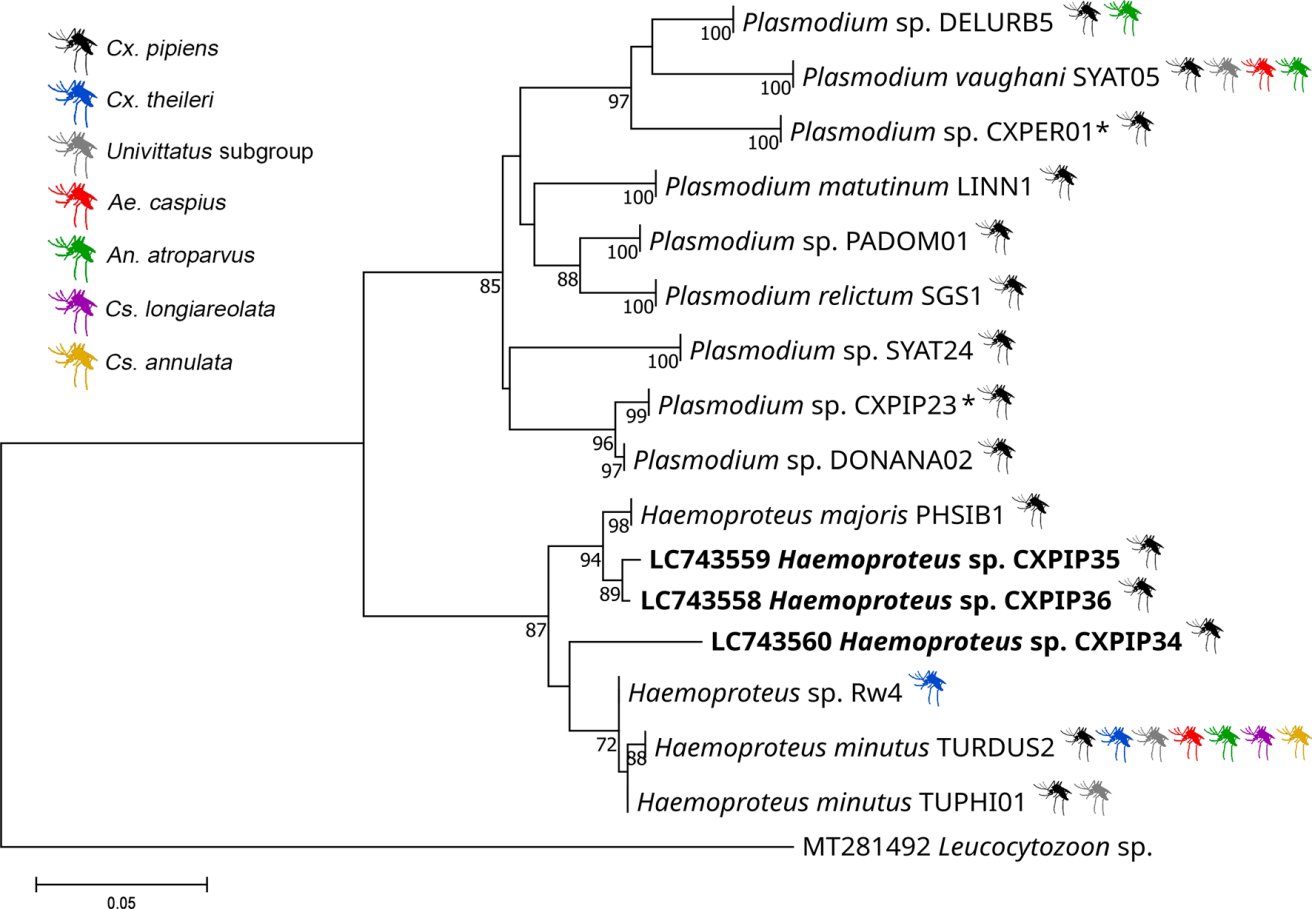
Maximum likelihood tree constructed using the GTR + G + I model for *Plasmodium* and *Haemoproteus* lineages. The analysis involved 30 nucleotide sequences (two sequences were collapsed into one for the same lineage with the exception of newly discovered lineages), resulting in a final dataset of 441 positions. The consensus tree probability was -1713.51. Support values for the branches were estimated using aLRT/Bootstrap with 1000 repetitions for each method. The size bar indicates 0.05 replacements per site. The sequence *Leucocytozoon* MT281492 served as the outgroup. New lineages detected are highlighted in bold. The analysis was performed on *IQtree*.^*^ Accession number refers to the lineage synonyms DONANA09 and DONANA10 as described in MalAvi (version 2.5.7, August 5, 2023), respectively

The phylogenetic tree comprised nine *Plasmodium* and seven *Haemoproteus* lineages and showed the presence of distinct clusters of *Plasmodium* (Fig. 3). The two newly identified *Haemoproteus* lineages, CXPIP35 and CXPIP36 (GenBank reference: LC743559 and LC743558, respectively), showed a highly supported phylogenetic relationship with *Haemoproteus majoris* PHSIB1, while the CXPIP34 (GenBank reference: LC743560) lineage did not cluster with any other lineages (Fig. 3).

### Seasonal distribution of haemosporidian infections

**Fig. 4:**
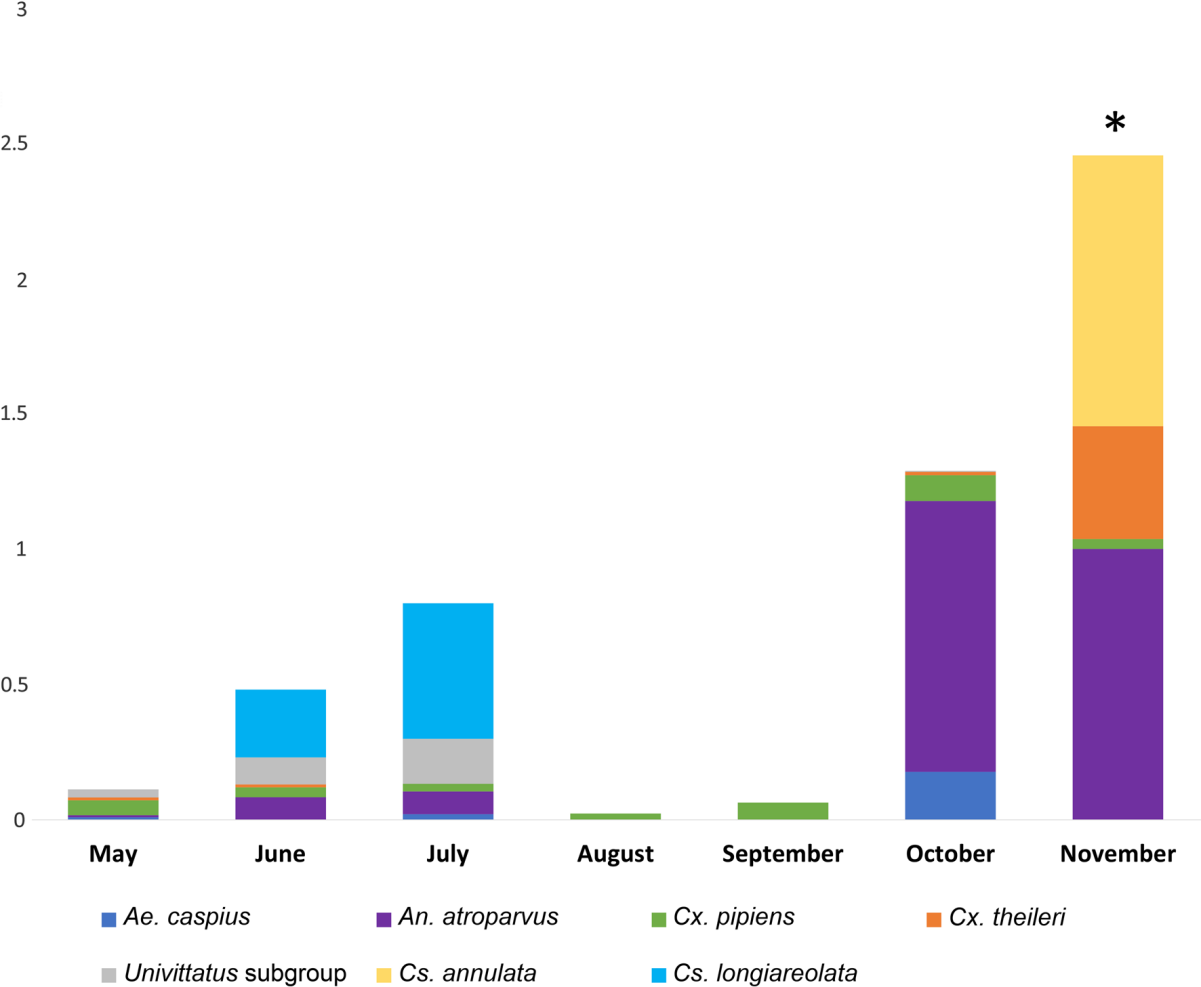
Mean minimum prevalence detected in the mosquito pools per month for the infected species screened for blood parasites. *Month with a significantly higher number of infected mosquitoes compared to all other months, as determined by the Chi-square test

The prevalence of avian malaria infections varied significantly across the months (*X*^*2*^ = 26.816; d.f. = 6; *P* < 0.001). Subsequent post hoc analysis using the Bonferroni method revealed that the prevalence of avian malaria parasites was significantly higher in November than in other months (*P* < 0.001; residuals = 4.704; Fig. 4).

### Feeding source identification in blood-fed mosquitoes

Of 5859 collected mosquitoes, only 27 were found to be blood-fed individuals (14 *Cx. pipiens majors*, two *Cx. pipiens molestus*, three *Cx. pipiens* hybrids, four *Cx. theileri*, and four *An. atroparvus*) (Table 3). The blood meal source was identified for all 27 engorged mosquitoes, with 52.6% of *Cx. pipiens* feeding on birds and the remaining 47.4% feeding on mammals. In contrast, all *Cx. theileri* and *An. atroparvus* were found to have fed on mammals. Interestingly, six of the 14 engorged *pipiens* forms fed on mammals (five fed on humans and one on dogs). A similar trend was observed in the *molestus* forms and hybrids, with no preference for feeding on both birds and mammals (Table 3).

**Table 3:** Total number of mosquitoes with blood meals from different vertebrate host species. For the *Culex pipiens* complex, the genetic subspecies is shown

Host feeding source	Vector species (subspecies)				
	*Anopheles atroparvus*	*Culex pipiens* (*pipiens*)	*Culex pipiens* (*molestus*)	*Culex* hybrids (*pipiens/molestus*)	*Culex theileri*
*Emberiza calandra*		1			
*Galerida cristata*				1 (1)	
*Gallus gallus*			1		
*Hirundo rustica*		1			
*Passer montanus*		1			
*Petronia petronia*		1 (1)			
*Sitta europaea*		1			
*Sylvia atricapilla*		1			
*Turdus merula*		2 (1)			
*Bos taurus*			1		1
*Canis lupus familiaris*		1			
*Homo sapiens*	4	5 (1)		2	3
Total	4	14	2	3	4

Number of mosquitoes carrying parasites is shown between brackets

## Discussion

Understanding the composition and phenology of the vector community, as well as its interaction with different lineages of haemosporidian parasites, is essential for comprehending the transmission dynamics of vector-borne pathogens. Traditionally, the study of transmission patterns of avian malaria parasites has mainly focused on investigating the relationships between vertebrate hosts and pathogens while largely overlooking the role of the vector identity. By providing new insights into the local circulation of different *Plasmodium* and *Haemoproteus* parasites in mosquitoes, our study expands the existing knowledge of molecular detection of different Haemosporidia reported previously by Ferraguti et al. [24] and Gutiérrez-López et al. [26] in southern Spain and by Ventim et al. [68] in Portugal.

The mosquito species collected in this study have been previously reported in the southwestern Europe region [24, 30, 68, 69]. Our results indicate that vector abundance was highest from May to July, declining in August and September, followed by a rebound in October before dropping significantly in November. Similar phenological patterns have been observed in other studies in temperate regions in the Northern Hemisphere. For instance, Ferraguti et al. [24] showed a notable increase in mosquito abundance during the summer months and a decrease in early autumn in southern Spain. In Portugal, Ventim et al. [68] reported a higher abundance of vectors during June and July than during autumn, a trend also described by Roiz et al. [70] in northern Italy. In western Switzerland, Lalubin et al. [71] showed that *Cx. pipiens* peaked in abundance from June to August before declining. Moreover, in Kansas (USA), Ganser and Wisely [72] reported an increase in mosquito abundance from May to June, followed by a decrease at the end of June and a subsequent increase in August, further supporting our outcomes. Local variations in mosquito abundance may be attributed to differences in environmental factors (e.g. microclimate, vegetation, land use) or the degree of anthropization [8, 73, 74] as well as intra-annual variation in climatic factors of the studied areas (generally, mosquito abundance is higher when rainfall and temperature are high) [75, 76] or landscape species-specific relationships related to the mosquitoes present in the territory [77].

Avian malaria prevalence was higher in *Ae. caspius*, *An. atroparvus*, *Cs. annulata* and *Cs. longiareolata* than in other vector species (Table 1). This high prevalence of avian malaria in *Ae. caspius* has also been recorded by Ferraguti et al. in southern Spain [24]. Nevertheless, this outcome may seem counterintuitive since *Ae. caspius* has been shown to mainly feed on mammals [39], and therefore a high prevalence of avian malaria parasites in this species would not be expected. This pattern could be explained by the lower relative abundance of mammals compared to birds in the area where the samples were collected. No infections were detected in *Ae. berlandi*, *Ae. echinus*, *Ae. pulcritarsis*, *Ae. vexans* and *Cs. subrochea*, which may be due to the low sample sizes of these species in this study.

Regarding the diversity of blood parasites detected, we found nine *Plasmodium* and eight *Haemoproteus* lineages. Six *Plasmodium* lineages (CXPER01, CXPIP23, DELURB5, DONANA02, SGS1 and SYAT05) had been previously detected in mosquitoes in southern Spain [24], while Veiga et al. [78] also detected TURDUS2 and SYAT05 in *Culicoides* species from Almeria. In turn, SGS1 and SYAT05 were also previously described in *Cx. pipiens* specimens in Barcelona [79] and in *Culex* mosquitoes from Portugal [68]. Moreover, six of the haemosporidian lineages we detected in mosquitoes have also been reported in resident birds in central and southern Spain over the past 15 years (*Plasmodium* DELURB5, LINN1, PADOM01, SGS1, SYAT05 and SYAT24), thus confirming local circulation of these parasites in the Iberian Peninsula [80–88].

We found that > 70% of the vector-lineage associations identified in this study had not been described in previous studies, thus representing new vector-parasite assemblages. This finding highlights the need for more vector-focused studies and efforts to better understand the dynamics of vector-parasite interactions. However, our methodology involved the analysis of full mosquito specimens (including head, thorax and abdomen). It is important to note that some haemosporidian parasites found in vectors may not fully mature to form sporozoites and lead to abortive infections, and hence the use of parasite DNA amplification via PCR techniques may present some limitations in the assessment of vector competence and vectorial capacity [20]. Results from further studies assessing successful sporogonic development and invasion of salivary glands by sporozoites are needed to supplement our findings and determine the competence of these vectors to transmit these haemosporidian lineages [16].

*Haemoproteus minutus* TURDUS2 and *P. vaughani* SYAT05 were the most generalist and abundant avian malaria lineages in our samples as they were found in seven and four mosquito species, respectively (Table 2, Fig. 3). Previous studies in birds have considered TURDUS2 and SYAT05 as haemosporidian generalist lineages [59, 89–91] as they were detected in multiple avian species. Notably, while SYAT05 has been described in six mosquito species in other studies, TURDUS2 was only reported in *Cx. pipiens* in previous studies [59], leading to a controversy over the definition of generalist parasite of these malaria lineages depending on whether they infect a vector or a vertebrate host. However, the outcomes from our study have revealed that these haemosporidian lineages are found in many vector species from different genera, thus confirming the generalist behavior of these malaria lineages in both vector and bird hosts.

Generalist parasites typically result in higher prevalence rates than more host-specific parasites, possibly because they can exploit a specific subset of hosts, leading to higher infection rates [87]. Our findings support this idea as we observed the highest prevalence in mosquitoes infected with these generalist haemosporidian lineages. However, it is important to note that the relationship between parasite specialization and prevalence can vary among different studies. For instance, in some cases, specialized avian Haemosporidia parasites may exhibit higher prevalence than generalists [92], while the opposite trend has also been observed [93]. Furthermore, parasite prevalence can be influenced by sample size, which, in turn, is dependent on environmental conditions. In our study, we identified other lineages that displayed more specialized behavior, being observed in only one mosquito species each. However, there were exceptions with *H. minutus* TUPHI1 and *Plasmodium* sp. DELURB5, which were found in two different mosquito species (*Cx. pipiens* and *Univittatus* subgroup, and *An. atroparvus* and *Cx. pipiens*, respectively).

Notably, *Plasmodium matutinum* LINN1, *P. relictum* SGS1 and *Haemoproteus* PHSIB1 were only detected in *Cx. pipiens* in our study, although they have been previously described in six, eight and one mosquito species, respectively. Moreover, these lineages have been considered generalist parasites in previous studies in birds [94–97]. These discrepancies may be explained by the higher relative abundance of *Cx. pipiens* in our study compared to other potential vectors. Although we found a high abundance of mosquitoes during late spring-early summer, our findings showed a maximum peak of avian malaria prevalence in vectors in October and November. This pattern has been previously reported in southern Spain [24] and Portugal [68]. A maximum prevalence of haemosporidians in vectors during autumn has been also described in Japan [23, 98]. However, other studies conducted in Turkey [99] and Switzerland [71] found higher infection prevalence in mosquitoes during the summer months. This observed trend in seasonally variation in avian malaria infection in vectors can be explained by the presence of haemosporidian lineages in suitable bird hosts. In this sense, Neto et al. [29] explored the seasonal variation of probability of infection by *Haemoproteus* and *Plasmodium* spp. in house sparrows from the same area as our study, showing a maximum prevalence of haemosporidian infection in late summer/early autumn. This difference in the time showing maximum peak of haemosporidian infection between avian hosts and vectors may be explained because the asexual cycle of haemosporidian parasites inside the bird hosts includes a time lapse between the release of sporozoites by vectors when taking a blood meal until the presence of micro and macrogametocytes in the avian bloodstream ready for parasite transmission to new vectors [12]. Additionally, *Plasmodium* parasitemia typically increases from February to September, peaking in autumn. This increases the likelihood of transmission of malaria lineages from infected birds with higher parasitemia to a greater number of mosquitoes during this season [100].

The unique characteristics of the studied area in the southwest of the Iberian Peninsula, in terms of biodiversity of insect vector and bird species, as well as the active circulation of pathogens in the environment, make this environment an ideal location for the study of vector-borne diseases [86, 87, 101–103]. However, our results can also be extrapolated to other Mediterranean regions, given that climate change forecasts predict an increase in temperatures in Spain, which will drastically alter the distribution of zoonotic mosquitoes [104].

The mammophilic feeding preference of both *An. atroparvus* and *Cx. theileri* species was confirmed [105, 106]. However, while *Cx. pipiens* is recognized as a primarily ornithophilic species [107], it can also feed on mammals when they are available [108]. This opportunistic feeding behavior has been observed in both *Cx. pipiens* forms and in *Cx. pipiens* x *molestus* hybrids [107]. The co-occurrence of both *Cx. pipiens* forms in urban surface habitats can result in a wide diversity of behavioral and ecological traits, making it difficult to categorize them [107]. This heterogeneous distribution has been observed in southern Europe [107, 109], contrasting with northern Europe, where harsh winters and strong divergent selection limit gene flow, allowing the two forms to maintain a remarkably divergent set of behaviors [107]. The diversity and heterogeneity of *Cx. pipiens* in mid-latitudes pose a challenge in accurately predicting their distribution and ecological behavior, which can potentially result in significant public health concerns. Indeed, these forms can act as a bridge for various zoonotic diseases between wild animals and human hosts [110, 111].

## Conclusions

To sum up, our study in the southwestern Iberian Peninsula has revealed several important findings. First, we have found a highly diverse mosquito community in the area, with variations in abundance throughout the seasons, peaking in early summer. Second, we have identified several different lineages of *Haemoproteus* and *Plasmodium* in mosquito vectors, including three newly recorded lineages, and we have described more than 70% of new associations between these parasites and mosquitoes. Third, we have observed a maximum peak of avian malaria prevalence in mosquito vectors during November, thus revealing active avian malaria transmission during mid-autumn in southern Europe. Finally, we have shown that the *Cx. pipiens* complex exhibits opportunistic feeding behavior, biting both birds and mammals. Our study highlights the importance of the relationship between avian malaria and different mosquito species as well as the effect of phenological factors and host feed preference. This information provides key steps to understanding disease transmission and may aid in identifying priority areas for pathogen surveillance and vector control measurements.

### Supplementary Information

**Additional file 1: Table S1**. Results of the estimated marginal means testing the relationships between mosquito abundance and the months of the sampling. Significant relationships (*p *≤ 0.05) are highlighted in bold.

## Data Availability

All the data presented in this manuscript are available from the corresponding author upon reasonable request.
