# Supplementary material for: Unravelling the mosquito-haemosporidian parasite-bird host network in the southwestern Iberian Peninsula: insights into malaria infections, mosquito community and feeding preferences
Source: Parasit Vectors. 2023 Nov 1;16:395. doi: 10.1186/s13071-023-05964-1 (PMC10619300; doi:10.1186/s13071-023-05964-1)
Supplement: Supplementary file 1 — Additional file 1: Table S1. Results of the estimated marginal means testing the relationships between mosquito abundance and the months of the sampling. Significant relationships (p ≤ 0.05) are highlighted in bold. [file 13071_2023_5964_MOESM1_ESM.docx]

**Table S1**. Results of the Estimated marginal means testing the relationships between mosquito abundance and the months of the sampling. Significant relationships (*p* ≤ 0.05) are highlighted in bold.

| **Contrast months** | **Estimate** | **S.E.** | ***t*** | ***p*** |
| --- | --- | --- | --- | --- |
| 5-6 | 0.639 | 1.61 | 0.398 | 0.999 |
| 5-7 | -3.284 | 1.33 | -2.465 | 0.175 |
| 5-8 | 4.137 | 2.46 | 1.682 | 0.628 |
| **5-9** | **7.735** | **2.02** | **3.826** | **0.003** |
| 5-10 | -1.087 | 1.64 | -0.663 | 0.994 |
| 5-11 | 7.670 | 3.42 | 2.244 | 0.275 |
| 6-7 | -3.923 | 1.58 | -2.476 | 0.171 |
| 6-8 | 3.497 | 2.60 | 1.343 | 0.831 |
| **6-9** | **7.096** | **2.20** | **3.231** | **0.022** |
| 6-10 | -1.727 | 1.85 | -0.933 | 0.967 |
| 6-11 | 7.031 | 3.52 | 1.995 | 0.420 |
| **7-8** | **7.421** | **2.45** | **3.034** | **0.041** |
| **7-9** | **11.019** | **2.01** | **5.495** | **<.001** |
| 7-10 | 2.197 | 1.62 | 1.357 | 0.824 |
| **7-11** | **10.954** | **3.41** | **3.213** | **0.024** |
| 8-9 | 3.598 | 2.88 | 1.249 | 0.874 |
| 8-10 | -5.224 | 2.63 | -1.990 | 0.423 |
| 8-11 | 3.533 | 3.99 | 0.886 | 0.975 |
| **9-10** | **-8.822** | **2.22** | **-3.972** | **0.002** |
| 9-11 | -0.065 | 3.73 | -0.017 | 1.00 |
| 10-11 | 8.757 | 3.54 | 2.474 | 0.172 |
